# Multi-Laboratory Validation of a Loop-Mediated Isothermal Amplification Method for Screening *Salmonella* in Animal Food

**DOI:** 10.3389/fmicb.2019.00562

**Published:** 2019-03-28

**Authors:** Beilei Ge, Kelly J. Domesle, Qianru Yang, Thomas S. Hammack, Shizhen S. Wang, Xiaohong Deng, Lijun Hu, Guodong Zhang, Yuan Hu, Xiaokuang Lai, Kyson X. Chou, Jan Ryan Dollete, Kirsten A. Hirneisen, Sammie P. La, Richelle S. Richter, Diyo R. Rai, Azadeh A. Yousefvand, Paul K. Park, Cindy H. Wu, Tameji Eames, David Kiang, Ju Sheng, Dancia Wu, Lori Hahn, Lisa Ledger, Cynthia Logie, Qiu You, Durda Slavic, Hugh Cai, Sherry L. Ayers, Shenia R. Young, Ruiqing Pamboukian

**Affiliations:** ^1^Division of Animal and Food Microbiology, Office of Research, Center for Veterinary Medicine, United States Food and Drug Administration, Laurel, MD, United States; ^2^Office of Regulatory Science, Center for Food Safety and Applied Nutrition, United States Food and Drug Administration, College Park, MD, United States; ^3^Office of Analytics and Outreach, Center for Food Safety and Applied Nutrition, United States Food and Drug Administration, College Park, MD, United States; ^4^Northeast Food and Feed Laboratory, Office of Regulatory Affairs, United States Food and Drug Administration, Jamaica, NY, United States; ^5^Pacific Southwest Food and Feed Laboratory, Office of Regulatory Affairs, United States Food and Drug Administration, Irvine, CA, United States; ^6^San Francisco Laboratory, Office of Regulatory Affairs, United States Food and Drug Administration, Alameda, CA, United States; ^7^Food and Drug Laboratory Branch, California Department of Public Health, Richmond, CA, United States; ^8^Office of Indiana State Chemist, Purdue University, West Lafayette, IN, United States; ^9^Animal Health Laboratory, University of Guelph, Guelph, ON, Canada; ^10^Office of Regulatory Science, Office of Regulatory Affairs, United States Food and Drug Administration, Rockville, MD, United States

**Keywords:** LAMP, *Salmonella*, multi-laboratory, validation, animal food

## Abstract

Loop-mediated isothermal amplification (LAMP) has gained wide popularity in the detection of *Salmonella* in foods owing to its simplicity, rapidity, and robustness. This multi-laboratory validation (MLV) study aimed to validate a *Salmonella* LAMP-based method against the United States Food and Drug Administration (FDA) *Bacteriological Analytical Manual* (BAM) Chapter 5 *Salmonella* reference method in a representative animal food matrix (dry dog food). Fourteen independent collaborators from seven laboratories in the United States and Canada participated in the study. Each collaborator received two sets of 24 blind-coded dry dog food samples (eight uninoculated; eight inoculated at a low level, 0.65 MPN/25 g; and eight inoculated at a high level, 3.01 MPN/25 g) and initiated the testing on the same day. The MLV study used an unpaired design where different test portions were analyzed by the LAMP and BAM methods using different preenrichment protocols (buffered peptone water for LAMP and lactose broth for BAM). All LAMP samples were confirmed by culture using the BAM method. BAM samples were also tested by LAMP following lactose broth preenrichment (paired samples). Statistical analysis was carried out by the probability of detection (POD) per AOAC guidelines and by a random intercept logistic regression model. Overall, no significant differences in POD between the *Salmonella* LAMP and BAM methods were observed with either unpaired or paired samples, indicating the methods were comparable. LAMP testing following preenrichment in buffered peptone water or lactose broth also resulted in insignificant POD differences (*P* > 0.05). The MLV study strongly supports the utility and applicability of this rapid and reliable LAMP method in routine regulatory screening of *Salmonella* in animal food.

## Introduction

*Salmonella* is a ubiquitous human and animal pathogen, with human outbreak-related illnesses broadly attributed to multiple food categories of plant and animal origins (Interagency Food Safety Analytics Collaboration [IFSAC], 2018). The presence of *Salmonella* in animal food (e.g., pet food, animal feed, and raw materials and ingredients) is also well documented (Li et al., 2012; Ge et al., 2013; Hsieh et al., 2014; Nemser et al., 2014; Food and Agriculture Organization [FAO]/World Health Organization [WHO], 2015; Jiang, 2016; Molina et al., 2016; Magossi et al., 2018), which impacts not only animal health but also human food safety due to consumption of animal-derived food or direct contact with pet food (Crump et al., 2002; Food and Agriculture Organization [FAO]/World Health Organization [WHO], 2015). The FDA Food Safety Modernization Act (FSMA) prioritizes preventive controls for human and animal foods, emphasizing vigilant product testing and environmental monitoring for zoonotic pathogens such as *Salmonella* (Food and Drug Administration [FDA], 2017a,b). Rapid and reliable methods are thus in great need to effectively support such efforts.

Current *Salmonella* testing in foods relies on microbiological culturing, which consists of time-consuming and labor-intensive procedures that require days or weeks for a definitive result (International Organization for Standardization [ISO], 2017; United States Department of Agriculture [USDA], 2017; Andrews et al., 2018). Rapid, sensitive, and specific nucleic acid amplification tests (NAATs), including PCR, real-time quantitative PCR (qPCR), and loop-mediated isothermal amplification (LAMP), have been developed and applied in the detection and identification of *Salmonella* in foods (Malorny et al., 2009; Balachandran et al., 2012; Lofstrom and Hoorfar, 2012; Cheng et al., 2015; Yang et al., 2016; Domesle et al., 2018; Hu et al., 2018). The isothermal LAMP method, in particular, has gained wide popularity as highlighted in a recent comprehensive review (Yang et al., 2018). Two distinct advantages of LAMP over PCR are running at a constant temperature (Notomi et al., 2000) and high tolerance to matrix inhibitors (Kaneko et al., 2007), which obviate the need for a sophisticated thermocycler or a complicated DNA extraction protocol. These attractive features have led to the development of many new *Salmonella* LAMP assays, portable microfluidic devices, and commercially available systems (Yang et al., 2018).

Validation plays a critical role in the life cycle of a method from development to implementation. Despite the growing enthusiasm in developing new *Salmonella* LAMP assays, limited effort has been devoted to validate the assay performance against well-established reference methods (International Organization for Standardization [ISO], 2017; United States Department of Agriculture [USDA], 2017; Andrews et al., 2018). These validation studies, performed at single laboratory, independent laboratory, and collaborative study (multi-laboratory) levels, represent rigorous evaluations of an alternative method’s performance compared with that of the reference method in a food matrix when conducted per international guidelines (Association of Official Analytical Chemists [AOAC], 2012; International Organization for Standardization [ISO], 2016). Similar FDA guidelines have been established for the validation of microbiological methods in foods (Food and Drug Administration [FDA], 2015). Methods that have successfully gone through multi-laboratory validation (MLV) are thus suitable for routine regulatory use. We previously developed a LAMP assay specifically targeting the *Salmonella* invasion gene *invA* and showed it to be rapid, reliable, and robust in multiple food matrices (Chen et al., 2011; Yang et al., 2013, 2014, 2015, 2016; Domesle et al., 2018). The method was 100% specific among 300 strains (247 *Salmonella* of 185 serovars and 53 non-*Salmonella*) tested and was capable of detecting <1 CFU/25 g in animal food (Domesle et al., 2018). Following FDA guidelines (Food and Drug Administration [FDA], 2015), we recently completed a stringent single-laboratory validation of the method in six animal food matrices including cattle feed, chicken feed, horse feed, swine feed, dry cat food, and dry dog food (Domesle et al., 2018).

This MLV collaborative study aimed to validate the *invA*-based *Salmonella* LAMP assay as performed on the Genie II or Genie III platform (OptiGene Ltd., West Sussex, United Kingdom) (alternative method) against the FDA BAM Chapter 5 *Salmonella* (reference method) in a representative animal food matrix (dry dog food) for future incorporation into the FDA’s compendium of analytical laboratory methods for food and feed safety. MLV participants included 14 independent collaborators from seven FDA, state, and academic laboratories in the United States and Canada. The MLV study also compared the effects of two preenrichment buffers used in LAMP and BAM on *Salmonella* detection in animal food.

## Materials and Methods

### Study Design

**FIGURE 1:**
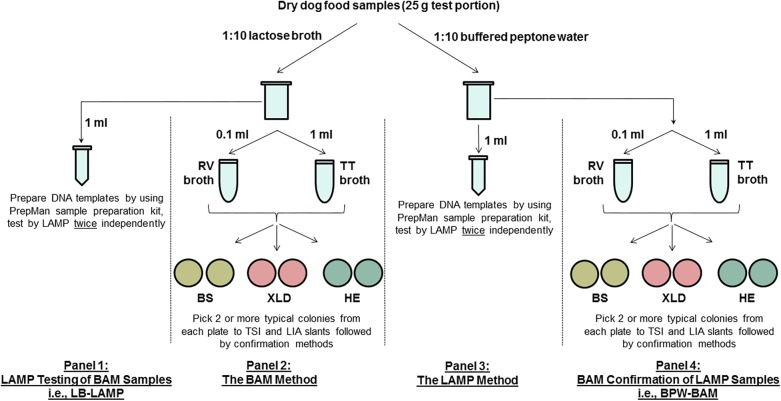
A schematic diagram of the MLV study design comparing the LAMP alternative method and the FDA BAM Chapter 5 reference method for the detection of *Salmonella* Infantis ATCC 51741 in 25 g dry dog food test portions. RV, Rappaport-Vassiliadis medium; TT, tetrathionate broth; BS, bismuth sulfite agar; XLD, xylose lysine desoxycholate agar; HE, Hektoen enteric agar; TSI, triple sugar iron agar; LIA, lysine iron agar.

Figure 1 shows a diagram of the MLV study design. The main component (panels 2 and 3) used an unpaired design, where different test portions were analyzed by the reference FDA BAM method (panel 2) and the alternative LAMP method (panel 3) following preenrichment in different buffers (lactose broth [LB] for BAM and buffered peptone water [BPW] for LAMP). All LAMP samples were confirmed by BAM culturing (panel 4, i.e., BPW-BAM). BAM samples were also tested by LAMP following LB preenrichment (panel 1, i.e., LB-LAMP), essentially forming paired samples (panels 1 and 2).

Fourteen independent collaborators (or independent teams), two each from seven FDA, state, and academic laboratories participated in the MLV. The FDA laboratories were from the Office of Regulatory Science at FDA’s Center for Food Safety and Applied Nutrition, and Northeast Food and Feed Laboratory, Pacific Southwest Food and Feed Laboratory, and San Francisco Laboratory at FDA’s Office of Regulatory Affairs. Other participants were the Food and Drug Laboratory Branch at California Department of Public Health, Office of Indiana State Chemist, and Animal Health Laboratory at University of Guelph (ON, Canada).

### Sample Inoculation, Storage, and Shipment

Inoculated samples were prepared by Q Laboratories (Cincinnati, OH, United States). Briefly, bulk dry dog food in kibble form was obtained from a local pet store and screened for the presence of *Salmonella* by the BAM Chapter 5 reference method (Andrews et al., 2018) and the iQ-Check *Salmonella* II Real-Time PCR detection kit (Bio-Rad, Hercules, CA, United States) to confirm negative results.

Dry dog food confirmed negative for *Salmonella* was separated into two sets and inoculated with a lyophilized culture of *Salmonella enterica* serovar Infantis ATCC 51741 at two target levels: a high level of *ca.* 2 to 5 CFU/25 g test portion and a low level of *ca.* 0.2 to 2 CFU/25 g test portion. An uninoculated control set (0 CFU/25 g test portion) was also included. After inoculation, the three sets of bulk samples were homogenized and held at room temperature for 2 weeks for aging to simulate storage. Replicate samples (5–10; 25 g each) from the two inoculated sets were evaluated at three time points (immediately after inoculation and homogenization, after 1 week of aging, and after 2 weeks of aging) by BAM and iQ-Check methods to verify the target levels and homogeneity.

On the day of shipment, a five-tube three-level most probable number (MPN) analysis was performed by evaluating 5 × 50 g replicates, 5 × 25 g replicates, and 5 × 10 g replicates to obtain final inoculation levels in the dry dog food sample sets. The samples were apportioned (25 g each), packaged, labeled (with randomized, blind-coded, three-digit numbers), and shipped overnight to the seven participating laboratories. For each laboratory, four sets of eight samples from each of the three inoculation levels were sent, along with two sets of samples from the uninoculated control set reserved for aerobic plate count (APC).

### Overview of Sample Analysis

**FIGURE 2:**
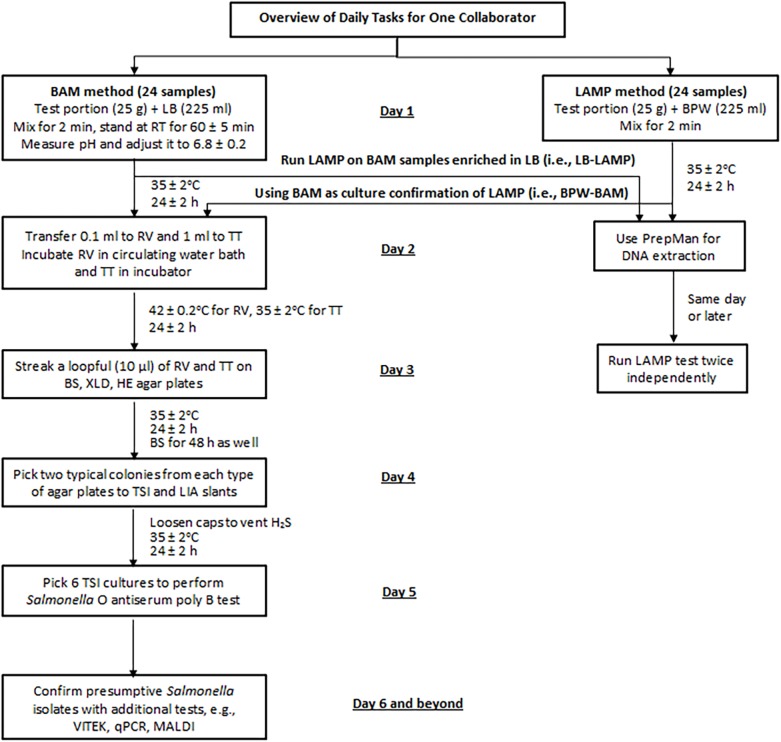
Sample analysis flowchart for the MLV study. LB, lactose broth; BPW, buffered peptone water; RV, Rappaport-Vassiliadis medium; TT, tetrathionate broth; BS, bismuth sulfite agar; XLD, xylose lysine desoxycholate agar; HE, Hektoen enteric agar; TSI, triple sugar iron agar; LIA, lysine iron agar; qPCR, real-time quantitative PCR; MALDI, Matrix Assisted Laser Desorption Ionization.

All collaborators (or teams) began testing on the same day. APC was performed by the pour plate method according to the FDA BAM Chapter 3 (Maturin and Peeler, 2018) or using the CompactDry plates (Hardy Diagnostics, Santa Maria, CA, United States). On day 1, each collaborator processed 24 samples following the BAM method and 24 samples following the LAMP method (Figure 2). Additionally, all LB preenrichment cultures from the BAM samples were tested by LAMP (i.e., LB-LAMP), and all BPW preenrichment cultures from the LAMP samples were processed with BAM for culture confirmation (i.e., BPW-BAM) from day 2. Therefore, a full data set from each collaborator consisted of 48 BAM and 48 LAMP results.

### The Loop-Mediated Isothermal Amplification (LAMP) Method

DNA extraction was performed by using the PrepMan Ultra sample preparation reagents (Thermo Fisher Scientific, Waltham, MA, United States). Briefly, aliquots (1 ml) of BPW or LB preenrichment cultures were first centrifuged at 900 × *g* for 1 min to remove large particles followed by another centrifugation at 16,000 × *g* for 2 min. The pellets were suspended in 100 μl of PrepMan Ultra reagent, heated at 100°C for 10 min, cooled to room temperature, and centrifuged again at 16,000 × *g* for 2 min. The supernatants (sample DNA extracts) were stored at -20°C until use.

**FIGURE 3:**
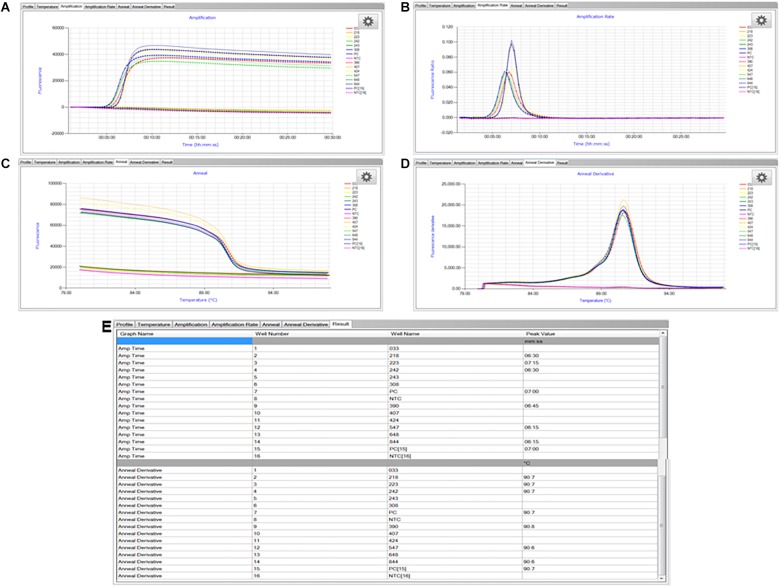
Representative LAMP graphs and results generated and viewed using the Genie Explorer software (version 2.0.6.3). **(A)** Amplification; **(B)** amplification rate; **(C)** anneal; **(D)** anneal derivative; and **(E)** results. Samples with three-digit codes correspond to dry dog food test portions with low-level inoculation of *Salmonella* Infantis ATCC 51741. Samples marked as PC are positive control, i.e., *S. enterica* Typhimurium ATCC 19585 (LT2) at 1.7 × 10^4^ CFU/reaction. Samples marked as NTC are no template control, i.e., molecular grade water.

The LAMP assay was carried out as described previously (Domesle et al., 2018). A positive control (*S. enterica* Typhimurium ATCC 19585 [LT2] at 1.7 × 10^4^ CFU/reaction) and no template control (molecular grade water) were included in each LAMP run. Briefly, the reagent mixture in a total volume of 25 μl contained 1× isothermal master mix ISO-001 (consisting of a strand-displacing *Gsp*SSD DNA polymerase large fragment from *Geobacillus* spp., thermostable inorganic pyrophosphatase, reaction buffer, MgSO_4_, dNTPs, and a double-stranded DNA binding dye; OptiGene Ltd.), 1× primer mix (0.1 μM each outer primer Sal4-F3 [GAACGTGTCGCGGAAGTC] and Sal4-B3 [CGGCAATAGCGTCACCTT], 1.8 μM each inner primer Sal4-FIP [GCGCGGCATCCGCATCAATATCTGGATGGTATGCCCGG] and Sal4-BIP [GCGAACGGCGAAGCGTACTGTCGCACCGTCAAAGGAAC], and 1 μM each loop primer Sal4-LF [TCAAATCGGCATCAATACTCATCTG] and Sal4-LB [AAAGGGAAAGCCAGCTTTACG]; Integrated DNA Technologies, Coralville, IA, United States), and 2 μl of sample DNA extract. The LAMP reaction was run at 65°C for 30 min followed by an annealing step from 98 to 80°C with 0.05°C decrement per second (Figure 3C) in the Genie II or Genie III real-time fluorometer (OptiGene Ltd.). Fluorescence readings were acquired using the 6-carboxyfluorescein (FAM) channel (Figure 3A) and time-to-peak values (*T_max_*; min) were determined when fluorescence ratios reached the maximum value of the amplification rate curve (Figure 3B). Corresponding annealing temperatures (*T_m_*; °C) of LAMP products were obtained in the anneal derivative curve (Figure 3D). Both *T_max_* and *T_m_* values were displayed in the “Results” tab at the end of the run (Figure 3E). Testing was repeated once independently.

### The Bacteriological Analytical Manual (BAM) Method

Procedures described in the BAM Chapter 5 (Andrews et al., 2018) were followed. All media and reagents were from BD Diagnostic Systems (Sparks, MD, United States) unless specified otherwise. As outlined in Figure 2, samples were processed by preenrichment in LB (day 1), selective enrichment in Rappaport-Vassiliadis (RV) medium and tetrathionate (TT) broth (day 2), selective plating on bismuth sulfite (BS) agar, xylose lysine desoxycholate (XLD) agar, and Hektoen enteric (HE) agar (day 3), biochemical confirmation on triple sugar iron agar (TSI) slant and lysine iron agar (LIA) slant (day 4), and serological identification by *Salmonella O* antiserum poly B (day 5). Additional confirmation tests performed included VITEK 2 Gram-negative biochemical identification method (AOAC *Official Method* 2011.17), Bruker MALDI Gram-negative Biotyper method (AOAC *Official Method* 2017.09) (Association of Official Analytical Chemists [AOAC], 2018), or real-time qPCR as specified in the BAM Chapter 5 (Andrews et al., 2018).

### Statistical Analysis

MPNs were calculated for the low- and high-level inoculated dry dog food using the LCF MPN calculator version 1.6 (Least Cost Formulations, Ltd., 2008). BAM samples were considered positive when *Salmonella* isolates were recovered. LAMP samples with the correct *T_m_* (approximately 90°C) and *T_max_* values between 5 and 30 min were considered positive. For this MLV, all LAMP testing results were reported as presumptive results (presumptive positive or presumptive negative). BAM and LAMP results for each contamination level (including uninoculated controls) were analyzed by using the probability of detection (POD) statistical model (Wehling et al., 2011) with the AOAC Binary Data Interlaboratory Study Workbook version 2.3 (Association of Official Analytical Chemists [AOAC], 2013). For each collaborator, PODs were calculated for the LAMP presumptive results (including false positive ones), LAMP confirmed by BAM results (including false negative ones), and LAMP final results (excluding false positive and false negative ones, i.e., only those LAMP samples tested positive by both LAMP and BAM confirmation), and the BAM reference results. LPOD values was determined by combining all valid collaborator-level POD data and the difference in LPOD (dLPOD) between two methods were calculated with a 95% confidence interval. The two methods were considered statistically significant when the dLPOD confidence interval did not contain zero.

Additionally, BAM and LAMP results were analyzed by using a random intercept logistic regression model for unpaired samples and Obuchowski’s modified McNemar’s test (Obuchowski, 1998) and a conditional logistic regression model for paired samples. Differences between the methods being compared were considered significant when *P* < 0.05.

## Results

The average APC was 2.1 × 10^1^ CFU/g (ranging from <1.0 × 10^1^ to 1.6 × 10^2^ CFU/g) for the uninoculated dry dog food controls. *Salmonella* MPNs obtained in the two inoculated sample sets, with a 95% confidence interval, were 0.65 MPN/25 g (0.30, 1.40) for the low level and 3.01 MPN/25 g (1.31, 6.89) for the high level. Two collaborators (9 and 10) mixed up sample bag sets among the four sets of samples received in their laboratory, resulting in uneven number (5–10) of samples tested per inoculation level. Nonetheless, the samples were not compromised as they were individually bagged and blindly coded, and their data were still included in the final statistical analysis for the MLV study. Data from another two collaborators (2 and 4) were excluded due to confirmed positive results among uninoculated controls. In total, there were 288 (12 collaborator × 24 samples/collaborator) data points each for LAMP and BAM in the final comparisons presented below, which include LAMP vs. BAM using unpaired samples, LAMP vs. BAM using paired samples, BPW vs. LB for use as LAMP preenrichment buffers, and BPW vs. LB for use as BAM preenrichment buffers.

### Unpaired Sample Statistical Analysis: LAMP Was Comparable to BAM

Table 1 shows the collaborator-level comparative results for the detection of *Salmonella* Infantis ATCC 51741 in 25 g dry dog food test portions by the LAMP alternative method versus the FDA BAM Chapter 5 reference method in an unpaired study design, i.e., different portions were analyzed by LAMP and BAM using different preenrichment buffers. For the uninoculated controls, collaborators 2, 4, and 5 had LAMP presumptive positive results with the rate as high as 75% for both collaborators 2 and 4. The single sample for collaborator 5 did not confirm positive by BAM culturing, but several samples for collaborators 2 and 4 did (LAMP final). Based on these results, data from collaborators 2 and 4 were excluded from the MLV. In addition, collaborator 1 had one LAMP presumptive negative sample confirming positive by BAM and collaborator 4 had one BAM sample testing positive by BAM. Fractional recovery (i.e., 25 to 75% positive responses) was obtained for the low inoculation level by all collaborators although 2, 4, 9, and 10 achieved that by only one method (LAMP presumptive or BAM reference). All high-level inoculated samples tested positive, regardless of the method used (Table 1).

**Table 1:** Comparative detection of *Salmonella* Infantis ATCC 51741 in 25 g dry dog food test portions by the LAMP method versus the FDA BAM Chapter 5 reference method in an unpaired study design.

Collaborator*^a^*	Number of positive samples/number of samples tested (probability of detection, POD) among dry dog food test portions*^b^*								
	
	Uninoculated control (0 MPN/25 g)			Low-level inoculation (0.65 MPN/25 g)			High-level inoculation (3.01 MPN/25 g)		
			
	LAMP presumptive	LAMP final	BAM reference	LAMP presumptive	LAMP final	BAM reference	LAMP presumptive	LAMP final	BAM reference
1	0/8 (0)	0/8 (0)*^c^*	0/8 (0)	4/8 (0.5)	4/8 (0.5)	6/8 (0.75)	8/8 (1)	8/8 (1)	8/8 (1)
2	6/8 (0.75)	1/8 (0.13)	0/8 (0)	7/8 (0.88)	5/8 (0.63)	6/8 (0.75)	8/8 (1)	8/8 (1)	8/8 (1)
3	0/8 (0)	0/8 (0)	0/8 (0)	6/8 (0.75)	6/8 (0.75)	2/8 (0.25)	8/8 (1)	8/8 (1)	8/8 (1)
4	6/8 (0.75)	2/8 (0.25)	1/8 (0.13)	8/8 (1)	6/8 (0.75)	5/8 (0.63)	8/8 (1)	8/8 (1)	8/8 (1)
5	1/8 (0.13)	0/8 (0)	0/8 (0)	4/8 (0.5)	3/8 (0.38)	6/8 (0.75)	8/8 (1)	8/8 (1)	8/8 (1)
6	0/8 (0)	0/8 (0)	0/8 (0)	4/8 (0.5)	4/8 (0.5)	5/8 (0.63)	8/8 (1)	8/8 (1)	8/8 (1)
7	0/8 (0)	0/8 (0)	0/8 (0)	2/8 (0.25)	2/8 (0.25)	6/8 (0.75)	8/8 (1)	8/8 (1)	8/8 (1)
8	0/8 (0)	0/8 (0)	0/8 (0)	5/8 (0.63)	5/8 (0.63)	4/8 (0.5)	8/8 (1)	8/8 (1)	8/8 (1)
9	0/10 (0)	0/10 (0)	0/6 (0)	4/5 (0.8)	3/5 (0.6)	5/11 (0.45)	9/9 (1)	9/9 (1)	7/7 (1)
10	0/6 (0)	0/6 (0)	0/10 (0)	3/9 (0.33)	3/9 (0.33)	7/7 (1)	9/9 (1)	9/9 (1)	7/7 (1)
11	0/8 (0)	0/8 (0)	0/8 (0)	6/8 (0.75)	6/8 (0.75)	5/8 (0.63)	8/8 (1)	8/8 (1)	8/8 (1)
12	0/8 (0)	0/8 (0)	0/8 (0)	4/8 (0.5)	4/8 (0.5)	2/8 (0.25)	8/8 (1)	8/8 (1)	8/8 (1)
13	0/8 (0)	0/8 (0)	0/8 (0)	5/8 (0.63)	5/8 (0.63)	4/8 (0.5)	8/8 (1)	8/8 (1)	8/8 (1)
14	0/8 (0)	0/8 (0)	0/8 (0)	4/8 (0.5)	3/8 (0.38)	6/8 (0.75)	8/8 (1)	8/8 (1)	8/8 (1)
All	13/112 (0.12)	3/112 (0.03)	1/112 (0.01)	66/110 (0.6)	59/110 (0.54)	69/114 (0.61)	114/114 (100)	114/114 (100)	110/110 (100)
Final*^d^*	1/96 (0.01)	0/96 (0)	0/96 (0)	51/94 (0.54)	48/94 (0.51)	58/98 (0.59)	98/98 (100)	98/98 (100)	94/94 (100)


*^a^ Collaborators 1 and 2 were from the same laboratory, so were collaborators 3 and 4, 5 and 6, 7 and 8, 9 and 10, 11 and 12, 13 and 14. *^*b*^* The LAMP presumptive column shows the results when LAMP samples were tested by LAMP (including false positive results). The LAMP final column shows the results when combining the LAMP presumptive and LAMP confirmed by BAM results (data not shown) to exclude false positive and false negative results, i.e., only LAMP samples tested positive by both LAMP and BAM confirmation were included. The BAM reference column shows the results when BAM samples were tested by BAM. *^*c*^* One sample was confirmed positive by BAM. The LAMP final result excluded this sample as it was negative by LAMP. *^*d*^* The Final row excluded data from collaborators 2 and 4 due to culture-confirmed positives in uninoculated test portions.*

Table 2 summarizes the statistics generated using the POD model and comparisons made using this model and a random intercept logistic regression model for unpaired samples (e.g., LAMP vs. BAM) and the Obuchowski’s modified McNemar’s test and a conditional logistic regression model for paired samples (e.g., LAMP presumptive vs. LAMP confirmed). For the low inoculation level, 51 out of 94 samples were LAMP presumptive positive (LPOD of 0.54) with 48 of them confirming positive (LPOD of 0.51). No false negative results were obtained (data not shown), therefore the LAMP final LPOD was also 0.51. Among 98 samples tested by BAM, 58 produced positive results (LPOD of 0.59). A dLPOD value of -0.08 with a 95% confidence interval (-0.24, 0.08) was obtained between LAMP final and BAM, indicating they were comparable. Similarly, for the high inoculation level and uninoculated controls, no significant differences were observed between LAMP final and BAM as confidence intervals for both dLPOD values contained zero. Based on dLPOD analysis, three other comparisons (i.e., LAMP presumptive vs. BAM, LAMP presumptive vs. LAMP confirmed, and LAMP presumptive vs. LAMP final) also showed no statistical significance. The statistical insignificance for all four comparisons at all three inoculation levels were separately confirmed by using aforementioned statistical models as indicated by *P*-values greater than 0.05 (Table 2).

**Table 2:** Summary of statistics generated using the POD model and other models for the detection of *Salmonella* Infantis ATCC 51741 in 25 g dry dog food test portions by the LAMP method versus the BAM reference method in an unpaired study design.

Parameter and comparisons	Combined POD and associated statistics (lower control limit, upper control limit) among dry dog food test portions		
	
	Uninoculated (0 MPN/25 g)	Low-level (0.65 MPN/25 g)	High-level (3.01 MPN/25 g)
Statistics generated using the POD model*^a^*			

LAMP presumptive positive/total number	1/96	51/94	98/98
LPOD	0.01 (0.00, 0.06)	0.54 (0.44, 0.65)	1.00 (0.96, 1.00)
*s*_r_	0.10 (0.09, 0.19)	0.51 (0.44, 0.54)	0.00 (0.00, 0.19)
*s*_L_	0.00 (0.00, 0.05)	0.00 (0.00, 0.23)	0.00 (0.00, 0.19)
*s*_R_	0.10 (0.09, 0.12)	0.51 (0.45, 0.54)	0.00 (0.00, 0.27)
*P*-value	0.4336	0.6048	1.0000
LAMP confirmed positive/total number	1/96	48/94	98/98
LPOD	0.01 (0.00, 0.06)	0.51 (0.41, 0.62)	1.00 (0.96, 1.00)
*s*_r_	0.10 (0.09, 0.19)	0.51 (0.44, 0.54)	0.00 (0.00, 0.19)
*s*_L_	0.00 (0.00, 0.05)	0.00 (0.00, 0.22)	0.00 (0.00, 0.19)
*s*_R_	0.10 (0.09, 0.12)	0.51 (0.45, 0.54)	0.00 (0.00, 0.27)
*P*-value	0.4336	0.6070	1.0000
LAMP final positive/total number	0/96	48/94	98/98
LPOD	0.00 (0.00, 0.04)	0.51 (0.41, 0.62)	1.00 (0.96, 1.00)
*s*_r_	0.00 (0.00, 0.19)	0.51 (0.44, 0.54)	0.00 (0.00, 0.19)
*s*_L_	0.00 (0.00, 0.19)	0.00 (0.00, 0.22)	0.00 (0.00, 0.19)
*s*_R_	0.00 (0.00, 0.27)	0.51 (0.45, 0.54)	0.00 (0.00, 0.27)
*P*-value	1.0000	0.6070	1.0000
BAM positive/total number	0/96	58/98	94/94
LPOD	0.00 (0.00, 0.04)	0.59 (0.47, 0.72)	1.00 (0.96, 1.00)
*s*_r_	0.00 (0.00, 0.19)	0.48 (0.41, 0.53)	0.00 (0.00, 0.19)
*s*_L_	0.00 (0.00, 0.19)	0.14 (0.00, 0.33)	0.00 (0.00, 0.19)
*s*_R_	0.00 (0.00, 0.27)	0.50 (0.44, 0.53)	0.00 (0.00, 0.27)
*P*-value	1.0000	0.0974	1.0000

Comparisons based on the POD model and other statistical models*^b^*			

dLPOD (LAMP final vs. BAM)	0.00 (-0.04, 0.04)	-0.08 (-0.24, 0.08)	0.00 (-0.04, 0.04)
dLPOD (LAMP presumptive vs. BAM)	0.01 (-0.03, 0.06)	-0.05 (-0.21, 0.11)	0.00 (-0.04, 0.04)
dLPOD (LAMP presumptive vs. LAMP confirmed)	0.00 (-0.05, 0.05)	0.03 (-0.12, 0.18)	0.00 (-0.04, 0.04)
dLPOD (LAMP presumptive vs. LAMP final)	0.01 (-0.03, 0.06)	0.03 (-0.12, 0.18)	0.00 (-0.04, 0.04)
*P*-value (LAMP final vs. BAM)	N/A	0.26	N/A
*P*-value (LAMP presumptive vs. BAM)	0.32	0.49	N/A
*P*-value (LAMP presumptive vs. LAMP confirmed)	1 (1)	0.99 (f)	N/A (N/A)
*P*-value (LAMP presumptive vs. LAMP final)	0.99 (f)	0.96 (f)	N/A (N/A)


*^*a*^ LPOD is a composite POD across collaborators and includes between-collaborator variation in addition to variation inherent in the binomial nature of the binary probabilities. *s*_*r*_ is repeatability standard deviation, *s*_*L*_ is among-collaborator standard deviation, *s*_*R*_ is reproducibility standard deviation. *P*-value is homogeneity test of collaborator PODs. *^*b*^* dLPOD is the difference in LPOD between two methods. The numbers in parenthesis are 95% confidence interval (lower control limit [LCL], upper control limit [UCL]) estimates on dLPOD. A confidence interval for dLPOD that does not contain 0 indicates a statistically significant difference between the two methods being compared. A random intercept logistic regression model was used for unpaired comparison and Obuchowski’s modified McNemar’s test and a conditional logistic regression model (numbers in parenthesis) were used for paired comparisons. f indicates that model fitting failed to converge. N/A, no test was done because of complete match of the results.*

### Paired Sample Statistical Analysis: LAMP Was Comparable to BAM

Table 3 shows the summary statistics for the LAMP and BAM methods when paired samples were used (LB-LAMP vs. BAM), i.e., the same test portions were analyzed by LAMP and BAM following preenrichment in LB. For the low inoculation level, 58 out of 98 samples were LB-LAMP positive (LPOD of 0.59) while 58 out of 98 samples were positive by BAM (LPOD of 0.59). Collaborator 13 reported one positive sample by LB-LAMP only, while collaborator 8 had one sample positive by BAM only (data not shown). A dLPOD value of 0.00 with a 95% confidence interval (-0.18, 0.18) was obtained, indicating the two methods were not significantly different. Similarly, for the high inoculation level and uninoculated controls, no significant differences were observed as confidence intervals for both dLPOD values contained zero. One uninoculated sample from collaborator 14 was positive by LB-LAMP but not BAM (data not shown). The statistical insignificance between LB-LAMP and BAM at all three inoculation levels was separately confirmed by using the Obuchowski’s modified McNemar’s test and the conditional logistic regression model (Table 3).

**Table 3:** Summary of statistics generated using the POD model and other models for the detection of *Salmonella* Infantis ATCC 51741 in 25 g dry dog food test portions by the LAMP method (with LB preenrichment) versus the BAM reference method in a paired study design.

Parameter and comparisons	Combined POD and associated statistics (lower control limit, upper control limit) among dry dog food test portions		
	
	Uninoculated (0 MPN/25 g)	Low-level (0.65 MPN/25 g)	High-level (3.01 MPN/25 g)
Statistics generated using the POD model*^a^*			

LB-LAMP positive/total number	1/96	58/98*^b^*	94/94
LPOD	0.01 (0.00, 0.06)	0.59 (0.46, 0.72)	1.00 (0.96, 1.00)
*s*_r_	0.10 (0.09, 0.19)	0.47 (0.41, 0.53)	0.00 (0.00, 0.19)
*s*_L_	0.00 (0.00, 0.05)	0.15 (0.00, 0.35)	0.00 (0.00, 0.19)
*s*_R_	0.10 (0.09, 0.12)	0.50 (0.44, 0.53)	0.00 (0.00, 0.27)
*P*-value	0.4336	0.0726	1.0000
BAM positive/total number	0/96	58/98*^b^*	94/94
LPOD	0.00 (0.00, 0.04)	0.59 (0.47, 0.72)	1.00 (0.96, 1.00)
*s*_r_	0.00 (0.00, 0.19)	0.48 (0.41, 0.53)	0.00 (0.00, 0.19)
*s*_L_	0.00 (0.00, 0.19)	0.14 (0.00, 0.33)	0.00 (0.00, 0.19)
*s*_R_	0.00 (0.00, 0.27)	0.50 (0.44, 0.53)	0.00 (0.00, 0.27)
*P*-value	1.0000	0.0974	1.0000

Comparisons based on the POD model and other statistical models*^c^*			

dLPOD (LB-LAMP vs. BAM)	0.01 (-0.03, 0.06)	0.00 (-0.18, 0.18)	0.00 (-0.04, 0.04)
*P*-value (LB-LAMP vs. BAM)	0.99 (f)	1 (1)	N/A (N/A)


*^*a*^ LPOD is a composite POD across collaborators and includes between-collaborator variation in addition to variation inherent in the binomial nature of the binary probabilities. *s*_*r*_ is repeatability standard deviation, *s*_*L*_ is among-collaborator standard deviation, *s*_*R*_ is reproducibility standard deviation. *P*-value is homogeneity test of laboratory (collaborator) PODs. *^*b*^* Collaborator 13 had one positive sample by LB-LAMP only, while collaborator 8 had one positive sample by BAM only. *^*c*^* dLPOD is the difference in LPOD between two methods. The numbers in parenthesis are 95% confidence interval (lower control limit [LCL], upper control limit [UCL]) estimates on dLPOD. A confidence interval for dLPOD that does not contain 0 indicates a statistically significant difference between the two methods being compared. Obuchowski’s modified McNemar’s test and a conditional logistic regression model (numbers in parenthesis) were used for LB-LAMP vs. BAM comparisons. f indicates that model fitting failed to converge. N/A, no test was done because of complete match of the results.*

### Preenrichment With BPW vs. LB Did Not Affect *Salmonella* Detection

Table 4 shows the statistics generated when unpaired samples were tested by either LAMP or BAM using different enrichment broths (LAMP vs. LB-LAMP and BPW-BAM vs. BAM). When tested by the LAMP method, 51 out of 94 low-level inoculated samples were positive (LPOD of 0.54) following BPW preenrichment, while 58 out of 98 samples produced positive results (LPOD of 0.59) following LB preenrichment. A dLPOD value of -0.05 with a 95% confidence interval (-0.22, 0.12) was obtained, indicating LAMP and LB-LAMP were not significantly different. Similarly, for the high inoculation level and uninoculated controls, no significant differences were observed as confidence intervals for both dLPOD values contained zero. Two different uninoculated samples were positive by either LAMP (for collaborator 5) or LB-LAMP (collaborator 14); neither was confirmed by BAM culturing (data not shown). The statistical insignificance at all three inoculation levels were separately confirmed by using the random intercept logistic regression model (Table 4). Therefore, preenrichment in BPW or LB did not significantly influence the LAMP results. The same held true for the BAM method when either BPW or LB were used as preenrichment buffers, i.e., there were no statistical significant differences for all three inoculation levels (Table 4).

**Table 4:** Summary of statistics generated using the POD model and other models for the detection of *Salmonella* Infantis ATCC 51741 in 25 g dry dog food test portions by LAMP or BAM when different preenrichment buffers were used for each one in an unpaired study design.

Parameter and comparisons	Combined POD and associated statistics (lower control limit, upper control limit) among dry dog food test portions		

	Uninoculated (0 MPN/25 g)	Low-level (0.65 MPN/25 g)	High-level (3.01 MPN/25 g)
Statistics generated using the POD model*^a^*			

LAMP positive/total number	1/96*^b^*	51/94	98/98
LPOD	0.01 (0.00, 0.06)	0.54 (0.44, 0.65)	1.00 (0.96, 1.00)
*s*_r_	0.10 (0.09, 0.19)	0.51 (0.44, 0.54)	0.00 (0.00, 0.19)
*s*_L_	0.00 (0.00, 0.05)	0.00 (0.00, 0.23)	0.00 (0.00, 0.19)
*s*_R_	0.10 (0.09, 0.12)	0.51 (0.45, 0.54)	0.00 (0.00, 0.27)
*P*-value	0.4336	0.6048	1.0000
LB-LAMP positive/total number	1/96*^b^*	58/98	94/94
LPOD	0.01 (0.00, 0.06)	0.59 (0.46, 0.72)	1.00 (0.96, 1.00)
*s*_r_	0.10 (0.09, 0.19)	0.47 (0.41, 0.53)	0.00 (0.00, 0.19)
*s*_L_	0.00 (0.00, 0.05)	0.15 (0.00, 0.35)	0.00 (0.00, 0.19)
*s*_R_	0.10 (0.09, 0.12)	0.50 (0.44, 0.53)	0.00 (0.00, 0.27)
*P*-value	0.4336	0.0726	1.0000
BPW-BAM positive/total number	1/96	48/94	98/98
LPOD	0.01 (0.00, 0.06)	0.51 (0.41, 0.62)	1.00 (0.96, 1.00)
*s*_r_	0.10 (0.09, 0.19)	0.51 (0.44, 0.54)	0.00 (0.00, 0.19)
*s*_L_	0.00 (0.00, 0.05)	0.00 (0.00, 0.22)	0.00 (0.00, 0.19)
*s*_R_	0.10 (0.09, 0.12)	0.51 (0.45, 0.54)	0.00 (0.00, 0.27)
*P*-value	0.4336	0.6070	1.0000
BAM positive/total number	0/96	58/98	94/94
LPOD	0.00 (0.00, 0.04)	0.59 (0.47, 0.72)	1.00 (0.96, 1.00)
*s*_r_	0.00 (0.00, 0.19)	0.48 (0.41, 0.53)	0.00 (0.00, 0.19)
*s*_L_	0.00 (0.00, 0.19)	0.14 (0.00, 0.33)	0.00 (0.00, 0.19)
*s*_R_	0.00 (0.00, 0.27)	0.50 (0.44, 0.53)	0.00 (0.00, 0.27)
*P*-value	1.0000	0.0974	1.0000

Comparisons based on the POD model and other statistical models*^c^*			

dLPOD (LAMP vs. LB-LAMP)	0.00 (-0.05, 0.05)	-0.05 (-0.22, 0.12)	0.00 (-0.04, 0.04)
dLPOD (BPW-BAM vs. BAM)	0.01 (-0.03, 0.06)	-0.08 (-0.24, 0.08)	0.00 (-0.04, 0.04)
*P*-value (LAMP vs. LB-LAMP)	1	0.49	N/A
*P*-value (BPW-BAM vs. BAM)	0.32	0.26	N/A


*^*a*^ LPOD is a composite POD across collaborators and includes between-collaborator variation in addition to variation inherent in the binomial nature of the binary probabilities. *s*_*r*_ is repeatability standard deviation, *s*_*L*_ is among-collaborator standard deviation, *s*_*R*_ is reproducibility standard deviation. *P*-value is homogeneity test of collaborator PODs. *^*b*^* Collaborator 5 had one positive sample by LAMP and collaborator 14 had one positive sample by LB-LAMP; neither were confirmed by BAM culturing. *^*c*^* dLPOD is the difference in LPOD between two methods. The numbers in parenthesis are 95% confidence interval (lower control limit [LCL], upper control limit [UCL]) estimates on dLPOD. A confidence interval for dLPOD that does not contain 0 indicates a statistically significant difference between the two methods being compared. A random intercept logistic regression model was used for LAMP vs. LB-LAMP and BPW-BAM vs. BAM comparisons. N/A, no test was done because of complete match of the results.*

## Discussion

This collaborative study rigorously validated a LAMP-based method for the screening of *Salmonella* in dry dog food at the multi-laboratory level. FDA’s current method validation guidelines for microbial pathogens in foods and feeds (Food and Drug Administration [FDA], 2015) were used, which align well with those from the AOAC and ISO (Association of Official Analytical Chemists [AOAC], 2012; International Organization for Standardization [ISO], 2016). In 2016, a United Kingdom study (D’Agostino et al., 2016) reported the validation of a LAMP/ISO 6579-based method for analyzing soya meal (an animal feed ingredient) for the presence of *S. enterica* in ten laboratories from eight European countries. For reasons of cost and logistics, that interlaboratory study did not use centrally prepared *Salmonella*-contaminated soya meal samples. Instead, commercially available certified *Salmonella* reference materials were used for inoculation by each participating laboratory, and no aging period was incorporated. Importantly, none of the three levels tested (0, 1–5, and 14–68 CFU per test portion) produced fractional positive results (25–75%) and three uninoculated control samples were confirmed positive for *Salmonella* (D’Agostino et al., 2016).

Unlike the United Kingdom study which was a “paired” trial, this MLV used an unpaired study design, i.e., different test portions from the same bulk samples inoculated centrally and aged for 2 weeks were analyzed by the LAMP alternative method and the BAM reference method using different preenrichment buffers. All LAMP samples were confirmed by BAM culturing and the reported LAMP final positive results were for samples tested positive by both LAMP and BAM confirmation. For the low-level inoculation, the overall proportions of positive responses were 51% for LAMP final and 59% for BAM (Table 2), which clearly satisfies the criteria outlined in validation guidelines of the AOAC, FDA, and ISO (Association of Official Analytical Chemists [AOAC], 2012; Food and Drug Administration [FDA], 2015; International Organization for Standardization [ISO], 2016). Multiple pairwise comparisons showed insignificant differences between LAMP and BAM by using either the POD analysis or other statistical models (Table 2), which highlights the success and rigor of this MLV study. Feedback from participating laboratories showed that the LAMP method was rapid, sensitive, practical, user-friendly, and easily adoptable.

A few false positive and/or false negative results were observed across the three testing levels in this MLV. For the low-level inoculated samples, there were three LAMP false positive results (one each for collaborators 5, 9, and 14 comparing LAMP presumptive and LAMP final) and no false negative results (Table 1 excluding data from collaborators 2 and 4). In all three cases, the *T_max_* values were rather high (>15 min) compared to others (*ca.* 7 min) (data not shown), indicating the amount of target DNA in the sample DNA extracts was low. This may be attributed to low contamination levels and dead or injured cells in these samples, which failed to reach the detection limit of BAM even after enrichments. The samples were shipped without dry ice since pet food is usually stored and shipped at ambient temperature; however, this may have contributed to some of the variability observed in the study. Another possibility is there was cross-contamination introduced during DNA extraction or assay setup for LAMP. For the uninoculated controls, one false positive (collaborator 5) and one false negative (collaborators 1, noted in footnote) results were observed. The former had high *T_max_* values (average of 14 min), while the latter was technically true negative (false positive by BAM) as it was an uninoculated control. Cross-contamination may have occurred when the two collaborators processed the samples for LAMP or BAM. Prior to this MLV, the LAMP assay has been extensively evaluated and high specificity (100% inclusivity and exclusivity) and high sensitivity (a detection limit of <1 CFU/25 g in animal food) have been demonstrated (Chen et al., 2011; Domesle et al., 2018; Yang et al., 2013, 2014, 2015, 2016). During the MLV study, none of the positive control samples produced false negative results, and none of no-template-control samples produced false positive results. These outcomes corroborate the high specificity and sensitivity of the *Salmonella* LAMP assay.

Besides the main component of the MLV study (unpaired design), we also compared the performance of LAMP and BAM using paired samples, i.e., the same test portions were analyzed by LAMP and BAM following LB preenrichment (LB-LAMP vs. BAM). One false positive (collaborator 13) and one false negative (collaborator 8) results were observed in low-level inoculated samples and one false positive was observed in one uninoculated sample (Table 3). Similar reasons described above may account for these false positive or false negative results observed. Nonetheless, paired samples also confirmed the statistically insignificant differences between the two methods using either POD analysis or other models.

As LAMP is gaining popularity in clinical diagnostics and food testing, many commercially available LAMP-based systems and assays have been developed and some were validated for *Salmonella* detection in food (Yang et al., 2018). These include the 3M Molecular Detection Assay (MDA) *Salmonella* (3M Food Safety, St. Paul, MN, United States) in raw ground beef and wet dog food (Bird et al., 2013, 2014), the 3M MDA 2 – *Salmonella* in raw ground beef and creamy peanut butter (Bird et al., 2016), and the SAS Molecular Tests *Salmonella* detection kit (SA Scientific Ltd., San Antonio, TX, United States) in ground beef, beef trim, ground turkey, chicken carcass rinses, bagged mixed lettuce, and fresh spinach (Bapanpally et al., 2014). In the two studies evaluating the 3M MDA *Salmonella* in wet dog food against the FDA BAM method, an unpaired study design was used and fractional positive results were obtained with POD analysis showing the methods were comparable (Bird et al., 2013, 2014). It is noteworthy that platforms used for the detection of LAMP amplicons were different in these studies as compared to our study. Bioluminescence was used for the 3M MDA assays, turbidity for the SAS kit, and fluorescence was used in our MLV (Genie II or Genie III). We previously tested the *Salmonella* LAMP assay on all three platforms (Chen et al., 2011; Yang et al., 2013, 2014, 2015, 2016; Domesle et al., 2018) and the fluorescence-based Genie II or Genie III was chosen for its simplicity, rapidity, portability, software interface, report format, and user-friendliness, with the annealing step offering an extra checkpoint to ensure the high specificity of the assay.

Another interesting outcome of the MLV study was the comparison of preenrichment buffers used for LAMP and BAM. We chose BPW as the default preenrichment buffer for the *Salmonella* LAMP method since preliminary data showed that shorter *T_max_* values were obtained for samples pre-enriched in BPW compared to those in LB (data not shown). Comparing *T_max_* values generated in this MLV for low- and high-level inoculated samples showed that BPW preenrichment generated *T_max_* values on average 1.9 min and 1.6 min shorter than those using LB preenrichment, suggesting the amount of DNA was higher when BPW was used as the preenrichment buffer. A recent study evaluating the 3M MDA *Salmonella* and the ANSR (stands for amplified nucleic single temperature reaction) detection system for *Salmonella* (Neogen Food Safety, Lansing, MI, United States) in egg products also showed that preenrichment in BPW improved the performance of both assays compared to LB (Hu et al., 2017). Nonetheless, using POD analysis for a qualitative method, statistically significant differences were not observed between BPW and LB for either LAMP or BAM, indicating they were comparable (Table 4).

It is worth noting that the *S.* Infantis ATCC 51741 used for inoculation in this MLV was a non-H_2_S producer with uncharacteristic serological reactions to *Salmonella* O antiserum poly B (data not shown). As a result, multiple confirmation methods besides serotyping were performed by participating laboratories, including VITEK 2, Bruker MALDI, and real-time qPCR, extending the time of sample testing by BAM to 2 weeks in contrast to 24 h by LAMP.

In our single-laboratory validation study (Domesle et al., 2018), five other animal food types (cattle feed, chicken feed, horse feed, swine feed, and dry cat food) besides dry dog food were successfully validated following FDA’s guidelines. The LAMP method validated in this MLB study in dry dog food should be applicable to these and other animal food types, per guidelines of the AOAC, FDA, and ISO (Association of Official Analytical Chemists [AOAC], 2012; Food and Drug Administration [FDA], 2015; International Organization for Standardization [ISO], 2016). Additional matrix extension studies may be readily performed in a variety of food matrices.

## Conclusion

In conclusion, the MLV study clearly demonstrated the utility and applicability of this rapid and reliable LAMP method in routine regulatory screening of *Salmonella* in animal food. As only LAMP-positive samples should continue with the isolation of *Salmonella* by the FDA BAM culture method, the LAMP method holds great potential to significantly reduce the time and labor and improve efficiency in animal food testing.

## Data Availability

All datasets generated for this study are included in the manuscript.

## Author Contributions

BG, KD, QRY, TH, and RP contributed to design and coordination of the study. BG, KD, and QRY performed the data analysis and interpretation. SW contributed to statistical analysis. XD, LJH, GZ, YH, XL, KC, JD, KH, SL, RR, DR, AY, PP, CW, TE, DK, JS, DW, LH, LL, CL, QY, DS, and HC contributed to sample testing and coordination of the study in seven participating laboratories. SA and SY assisted with sample analysis in the coordinating laboratory. All authors reviewed the final manuscript.

## Conflict of Interest Statement

The authors declare that the research was conducted in the absence of any commercial or financial relationships that could be construed as a potential conflict of interest.
